# Does Etelcalcetide reverse myelofibrotic bone changes due to hyperparathyroidism? A case report

**DOI:** 10.3389/fmed.2022.1050062

**Published:** 2023-02-23

**Authors:** Vincenzo Antonio Panuccio, Rocco Tripepi, Adele Postorino, Bruna Greve, Elena Sabattini, Esther Natalie Oliva

**Affiliations:** ^1^Nephrology, Dialysis and Transplantation Unit, Grande Ospedale Metropolitano Bianchi-Melacrino-Morelli, Reggio Calabria, Italy; ^2^Institute of Clinical Physiology, National Research Council, Reggio Calabria, Italy; ^3^Hematology Unit, Grande Ospedale Metropolitano Bianchi-Melacrino-Morelli, Reggio Calabria, Italy; ^4^Sezione Emolinfopatologica, Istituto di Ematologia “Seragnoli”, Policlinico S. Orsola, Bologna, Italy

**Keywords:** myelofibrosis, bone change, hyperparatiroidism, Etelcalcetide, dialysis (ESKD)

## Abstract

Secondary hyperparathyroidism (SHPT) in dialysis is common. A young man on chronic hemodialysis with SHPT developed pancytopenia with resistant anemia requiring transfusions. A bone marrow biopsy showed grade 3 fibrosis, depleted cellularity, osteosclerosis, and decreased myelopoiesis. He initiated Etelcalcetide 7⋅5 mg 3 times weekly with improvement in SHPT concomitant with near normalization of blood counts. Marrow biopsy at 12 months showed clearance of marrow reticulin, improvement of osteosclerosis and normalization of bone trabeculae, cellularity and myelopoiesis. This is a unique case in which Etelcalcetide treatment is comparable to parathyroidectomy on SHPT and is associated with significant improvement in severe myelofibrosis.

## Summary

Secondary hyperparathyroidism (SHPT) in dialysis is common. A young man on chronic hemodialysis with SHPT developed pancytopenia with resistant anemia requiring transfusions. A bone marrow biopsy showed grade 3 fibrosis, depleted cellularity, osteosclerosis, and decreased myelopoiesis. He initiated Etelcalcetide 7⋅5 mg 3 times weekly with improvement in SHPT concomitant with near normalization of blood counts. Marrow biopsy at 12 months showed clearance of marrow reticulin, improvement of osteosclerosis and normalization of bone trabeculae, cellularity and myelopoiesis. This is a unique case in which Etelcalcetide treatment is comparable to parathyroidectomy on SHPT and is associated with significant improvement in severe myelofibrosis.

## Introduction

Secondary hyperparathyroidism (SHPT) in dialysis patients is a common complication characterized by parathyroid gland hyperplasia resulting from resistance to parathyroid hormone (PTH). The clinical manifestations of secondary hyperparathyroidism include bone and joint pain and limb deformities (1). Goals of treatment are: serum phosphate levels maintained between 3⋅5 and 5⋅5 mg/dL (1⋅13 to 1⋅78 mmol/L); serum corrected total calcium levels maintained < 9⋅5 mg/dL (< 2⋅37 mmol/L); and PTH values maintained > 2 to < 9 times the upper limit (2). Only a minority of patients achieves the targets (so that treatment still remains a challenge. Refractory hyperparathyroidism occurs in approximately 7 to 10 per 1000 patient-years (3).

The role of SHPT on survival (4), bone fracture (5), cardiovascular events and comorbidities (6) is well described. We describe a case of SHPT on chronic hemodialysis treatment presenting with severe anemia. The association between anemia and SHPT remains largely unclarified (7).

## Case

**FIGURE 1:**
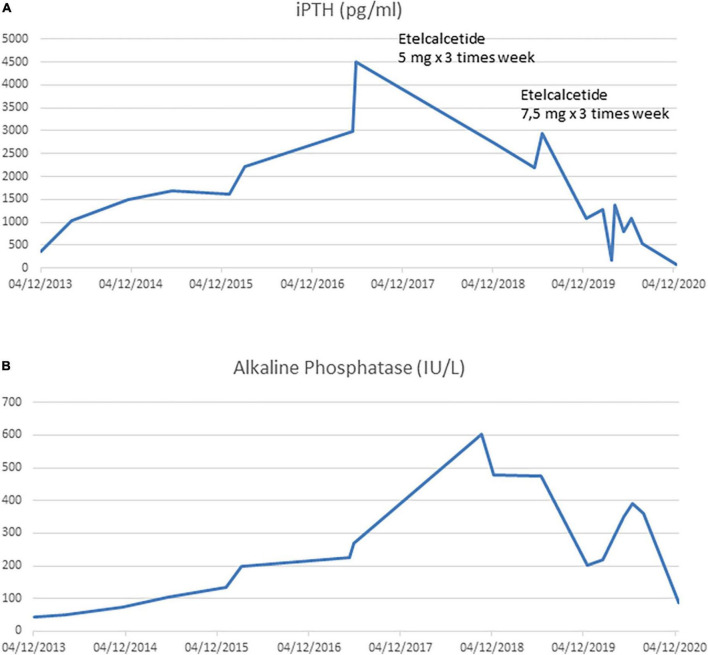
**(A)** Intact PTH trend during kidney graft dysfunction period and during hemodialysis period. **(B)** Alkaline Phosphatase trend during kidney graft dysfunction period and during hemodialysis period.

A 21-year-old man with a diagnosis of Autosomal Recessive Polycystic Kidney Disease and concomitant Caroli disease reached a final stage of chronic kidney disease (CKD) and started hemodialysis (HD) treatment. After 3 years, he underwent a kidney transplant from a cadaveric donor. His transplanted kidney worked well until the patient turned 31 years of age, when he developed rapid graft dysfunction (serum creatinine from 2⋅7 to 5 mg/dL). There was a concomitant increase in serum phosphate levels (8⋅3 mg/dl) and iPTH levels that progressively increased to 1,032 pg/ml despite a traditional therapy (vitamin D supplements, calcium-based phosphate binders). The laboratory changes over time are shown in Figure 1.

At age 32, the patient re-initiated HD treatment thrice weekly (dialysate calcium was stable 1.5 mmol/L) with a concomitant a progressive worsening of symptomatic hyperparathyroidism with bone pain. Cinacalcet at a dosage of 30 mg daily treatment was initiated and increased gradually to 120 mg daily without any benefit. Two years later, the clinical situation did not improve with a further enlargement of parathyroid glands; since the patient was not fully adherent to the therapy, a parathyroidectomy (PTX) was recommended.

**FIGURE 2:**
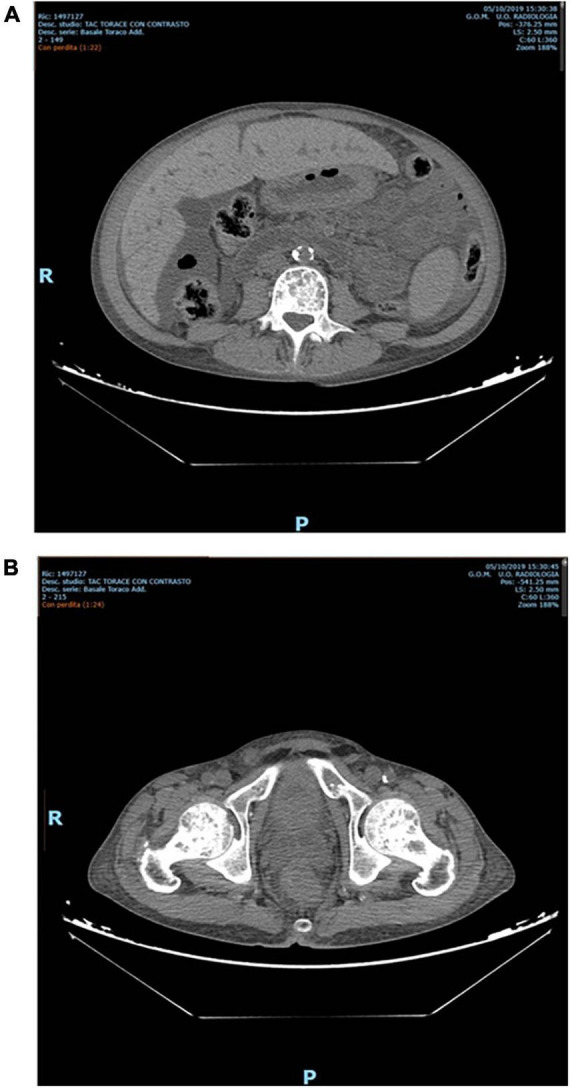
**(A)** Osteo-thickening areas affecting the entire spine. **(B)** Osteo-thickening areas in the pelvis.

Nevertheless, PTX was not performed for several reasons, amongst which patient’s refusal. Furthermore, after one year, despite standard erythropoietic stimulating agent (ESA) therapy, he developed severe anemia that required regular red blood cell transfusions. Intact PTH (iPTH) increased to 4500 pg/mL (Figure 1A) with a parallel rise in alkaline phosphatase > 600 UI/L (Figure 1B). A Computed Tomography scan showed multiple bone-thickening lesions (Figure 2). He thus initiated Etelcalcetide 5 mg intravenous 3 times a week after the hemodyalisis session without any benefit. The dosage was then increased to 7⋅5 mg but the patient gradually became frail and developed pancytopenia, low-grade fever and severe malnutrition.

**FIGURE 3:**
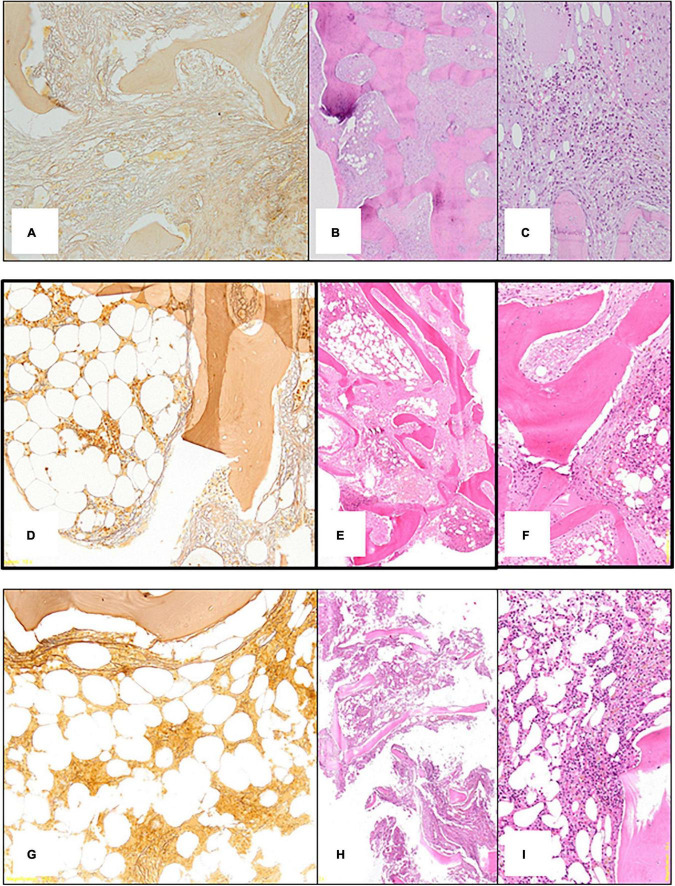
**(A)** First bone marrow biopsy: silver impregnation staining (x10) shows diffuse severe (grade 3) fibrosis. **(B)** First bone marrow biopsy: Hematoxylin Eosin (x10) shows depleted cellularity and frank osteosclerosis with bone thickening and remodeling. **(C)** First bone marrow biopsy: Hematoxylin Eosin (x10) shows residual decreased myelopoiesis. **(D)** Second bone marrow biopsy: silver impregnation staining (x10) shows variability of marrow fibrosis (severe 3 on the right side; moderate on the left side). **(E)** Second bone marrow biopsy: Hematoxylin Eosin (x10) shows mild improvement of the osteosclerosis. **(F)** Second bone marrow biopsy: Hematoxylin Eosin (x10) shows residual decreased myelopoiesis. **(G)** Third bone marrow biopsy: silver impregnation staining (x10) shows almost normalization of marrow fibrosis. **(H)** Third bone marrow biopsy: Hematoxylin Eosin (x10) shows clear-cut improvement of the osteosclerosis with normal appearing bone trabeculae. **(I)** Third bone marrow biopsy: Hematoxylin Eosin (x10) shows regular cellularity and myelopoiesis.

Due to the persistent cytopenia (Red Blood Cells 2⋅15 10^6/mmc, Hemoglobin 6⋅4 gr/dl, Total Leucocytes count 1.500 cells/mmc and Platelet 94.000 cells/mmc), hematological consultation was performed with bone marrow evaluation. Bone marrow histology showed diffuse severe grade 3 fibrosis (Figure 3A), depleted cellularity and osteosclerosis with bone thickening and remodeling (Figure 3B), and residual decreased myelopoiesis (Figure 3C). Blood samples for mutations in JAK-2, CALR, and MPL and BCR-ABL rearrangement were negative. There was no evidence for a myeloproliferative neoplasm (MPN) or metastatic lesions.

During the following months, while on a 7⋅5 mg dose of Etalcalcetide, there was a gradual reduction in iPTH (Figure 1A) and serum alkaline phosphatase (Figure 1B), up to 500 pg/dl and 200 IU/L, respectively. The patient developed asymptomatic, often severe, hypocalcemia (serum Calcium 3⋅4 mEq/L) which was managed with therapy (Table 1).

**TABLE 1:** Evolution of biomarkers and treatments changes over time.

	CKD stage 5	Chronic renal allograft dysfunction	Restart haemodialysis treatment	Values of biomarkers concomitant with the maximum iPTH levels recorded			Etelcalcetide 15 mg/week (start of therapy)	Intermediate evaluations			Etelcalcetide 22.5 mg/ week	First bone marrow biopsy	Intermediate evaluation	Second bone marrow biopsy	Intermediate evaluation	Third bone marrow biopsy	Last evaluation
Serum intact PTH (pg/ml)	361	1032	1009	2226	4496	2840	2643	2720	2187	2941		1086	1362	1084	519		73
Serum Calcium (mEq/L)	4.75	4.6	4.4	3.35	4.35	3.8	4.05	3.5	3.55	3.9	3.75	3.45	3.3	3.7	3.6	4.4	5.2
Phosphorus (mg/dl)	5.7	5.5	5.4	5.7	6.5	5.8	5.7	5	4.4	4.3	4.5	2.8	4.7	6.1	4.1	4.6	4.9
Alkalin Phosphatases (U.I./L)	43	51	51	197	270	348		478		474		202		392	359		88
Hemoglobin (gr/dl)	10.6	11.0	10.2		10.8	11.3	11.3	10.4	9.8	9.7	8.7	6.4	9.2	12.2	11.6	11.7	11.6
White blood cell (mm^3^)	6.4	7.3	4.59		4.37	2.74	3.9	2.63	3.63	3.63	3.48	1.49	2.33	3.83	3.44	3.33	6.36
Platelets (mm^3^)	186	161	159		113	117	123	123	107	92	127	120	146	107	113	119	152
Ferritin (ng/ml)	198		124		424			872		1520		2950		1640			1420
TSAT (%)	26.41		15.03		41.08	32.38		44.96		28		33.76		100.03			74.63
**Therapy**																	
	**CKD stage 5**	**Chronic renal allograft dysfunction**	**Restart haemodialysis treatment**	**Values of biomarkers concomitant with the maximum iPTH levels recorded**			**Etelcalcetide 15 mg/week (start of therapy)**	**Intermediate evaluations**			**Etelcalcetide 22.5 mg/week**	**First bone marrow biopsy**	**Intermediate evaluation**	**Second bone marrow biopsy**	**Intermediate evaluation**	**Third bone marrow biopsy**	**Last evaluation**
	**Tacrolimus** 9 mg/die	**Tacrolimus** 10 mg/die															
	**Azathioprine** 75 mg/die	**Azathioprine** 50 mg/die															
	**Prednisone** 5 mg/die																
	**Sodium polystynesulphonate** a spoon/die																
	**Fish-oil** 1000 mg/die																
	**Atorvastatin** 20 mg/die																
	**Folid acid** 400 mcg/die																
	**Atenolol** 100 mg/die				**Atenolol** 50 mg/die		**Atenolol** 25 mg/die		**Atenolol** 12.5 mg/die								
	**Amlodipine** 10 mg/die												**Amlodipine** 10 mg/die				
	**Telmisartan** 80 mg/die																
				**Doxazosin** 4 mg/die	**Doxazosin** 6 mg/die		**Doxazosin** 8 mg/die				**Doxazosin** 4 mg/die						
					**Nifedipine** 120 mg/die						**Nifedipine** 60 mg/die						
	**Esomeprazole** 40 mg/die								**Pantoprazole** 40 mg/die				**Lanzoprazole** 30 mg/die				
	**Darbepoetin alfa** 20 mcg every 3 week		**Darbepoetin alfa** 20 mcg every 10 days	**Epoietin alfa** 8000 U.I. week	**Epoietin alfa** 24000 U.I. week								**Epoietin alfa** 30.000 U.I. week	**Epoietin alfa** 4000 U.I. week			
				**Sodium iron gluconate** 62.5 mg week	**Sodium iron gluconate** 125 mg week												
							**Cianocobalamin** 3 mg week										
							**Etelcalcetide** 15 mg/week					**Etelcalcetide** 22.5 mg/week					
		**Sevelamer** 3200 mg/die		**Sevelamer** 4800 mg/die							**Sevelamer** 7200 mg/die	**Sevelamer** 4800 mg/die				**Sevelamer** 4800 mg/die	
			**Cinacalcet** 30 mg/die	**Cinacalcet** 90 mg/die	**Cinacalcet** 120 mg/die	**Cinacalcet** 120 mg/die											
	**Calcium Carbonate** 1000 mg/die				**Calcium Carbonate** 2000 mg/die								**Calcium Carbonate** 875 mg/die **+ Calcium gluconate lactate** 1.13 mg/die				
	**Calcitriol** 0.5 mcg/die						**Calcitriol** 0.5 mcg/die			**Calcitriol** 0.50 mcg alternate days			**Calcitriol** 0.50 mcg 5 days/week	**Calcitriol** 0.5 mcg/die			

The patient’s clinical condition gradually improved, and he no longer required transfusions (after 4 months Hb reached 11⋅4 gr/dl) and treatment with recombinant erythropoiesis stimulating agent was also reduced. At 6 months, bone marrow histology showed variable reduction of marrow fibrosis (Figure 3D, grade 2 on the left side and grade 3 on the right) improvement of osteosclerosis (Figure 3E) and only residual decreased myelopoiesis (Figure 3F). During the next 6 months the patient’s clinical conditions and anemia further improved. A bone marrow biopsy was repeated at 12 months and showed near normalization of marrow reticulin (fibrosis) (Figure 3G), clear-cut improvement of the osteosclerosis with normal appearance of bone trabeculae (Figure 3H), and regular cellularity and myelopoiesis (Figure 3I). Then Etelcalcetide dosage was reduced because iPTH was suppressed (iPTH 73 pg/ml) while serum calcium and phosphate levels were within normal limits. Remarkably, bone pain significantly reduced.

The evolution of biomarkers and treatment changes over time are given in Table 1.

## Discussion

Myelofibrosis secondary to renal osteodystrophy is an uncommon complication, rarely reported and usually associated with primary hyperparathyroidism (8). Marrow fibrosis and pancytopenia is related to the excessive iPTH that upregulates production of cytokines and paracrine factors in the bone marrow (IL-1a, IL-6, FNF-a, TGF–b, and platelet-derived growth factor) (9) and it has an important stimulatory effect on fibroblast proliferation. It is known that surgical parathyroidectomy is associated with a reduction of bone marrow fibrosis in primary hyperparathyroidism (10). This is the first case of tertiary hyperparathyroidism in which the effect of Etelcalcetide is comparable to parathyroidectomy on calcium-phosphate balance concomitant to a significant improvement in severe bone marrow fibrosis, bone structure and blood counts. The findings emerged in this case report depends on both the action of Etelcacetide *per se* (11) as well as the administration rout that guarantees an optimal adherence (12). Further investigation is needed to clarify the efficacy of Etelcalcetide on bone structure and fractures, effects on bone marrow and improvement of peripheral cytopenias in this frail population.

## Data availability statement

The datasets presented in this article are not readily available because Data collected for this paper were derived from clinical records, thus not available for sharing. Requests to access the datasets should be directed to VP, enzopanuccio@gmail.com.

## Ethics statement

Ethical review and approval was not required for the study on human participants in accordance with the local legislation and institutional requirements. The patients/participants provided their written informed consent to participate in this study. Written informed consent was obtained from the individual(s) for the publication of any potentially identifiable images or data included in this article.

## Author contributions

VP, AP, RT, and EO contributed to the study design and the drafting of the manuscript. VP, RT, BG, and ES contributed to data collection and interpretation of clinical aspects. All authors approved the final version of the manuscript.
